# Cardiovascular outcomes after simultaneous pancreas kidney transplantation compared to kidney transplantation alone: a propensity score matching analysis

**DOI:** 10.1186/s12882-021-02522-8

**Published:** 2021-10-21

**Authors:** U. G. Lange, S. Rademacher, B. Zirnstein, R. Sucher, K. Semmling, P. Bobbert, A. A. Lederer, D. Buchloh, L. Seidemann, D. Seehofer, N. Jahn, H.-M. Hau

**Affiliations:** 1 University Hospital Leipzig, Clinic of Visceral, Transplant, Thoracic and Vascular Surgery, Liebigstrasse 20, 04103 Leipzig, Saxony Germany; 2 Sana Hospital Borna, Clinic of Anaesthesia, Intensive Care and Palliative Medicine, Rudolf-Virchow-Strasse 2, 04552 Borna, Saxony Germany; 3 Ev. Hubertus Hospital Berlin, Clinic of Internal Medicine and Angiology, Spanische Allee 10-14, 14129 Berlin, Berlin, Germany; 4 Department of Anesthesiology and Intensive Care Medicine, University Hospital of Leipzig, Liebigstrasse 20, 04103 Leipzig, Germany; 5 Department of Visceral, Thoracic and Vascular Surgery, University Hospital and Faculty of Medicine Carl Gustav Carus, Technische Universität Dresden, Dresden, Germany

**Keywords:** Cardiovascular outcomes, Simultaneous pancreas kidney transplant, Kidney transplant alone, Insulins-dependent diabetes mellitus, Metabolic function, Glycometabolic control, Survival, Left ventricular function, Echocardiographic changes

## Abstract

**Background:**

Coronary heart disease due to arteriosclerosis is the leading cause of death in type 1 diabetic patients with end-stage renal disease (ESRD). The aim of this study was to evaluate the effect of simultaneous pancreas kidney transplantation (SPKT) compared to kidney transplantation alone (KTA) on survival, cardiovascular function and metabolic outcomes.

**Methods:**

A cohort of 127 insulin-dependent diabetes mellitus (IDDM) patients with ESRD who underwent either SPKT (*n* = 100) or KTA (*n* = 27) between 1998 and 2019 at the University Hospital of Leipzig were retrospectively evaluated with regard to cardiovascular and metabolic function/outcomes as well as survival rates. An additional focus was placed on the echocardiographic assessment of systolic and diastolic cardiac function pretransplant and during follow-up. To avoid selection bias, a 2:1 propensity score matching analysis (PSM) was performed.

**Results:**

After PSM, a total of 63 patients were identified; 42 patients underwent SPKT, and 21 patients received KTA. Compared with the KTA group, SPKT recipients received organs from younger donors (*p* < 0.05) and donor BMI was higher (*p* = 0.09). The risk factor-adjusted hazard ratio for mortality in SPKT recipients compared to KTA recipients was 0.63 (CI: 0.49–0.89; *P* < 0.05). The incidence of pretransplant cardiovascular events was higher in the KTA group (KTA: *n* = 10, 47% versus SPKT: *n* = 10, 23%; *p* = 0.06), but this difference was not significant. However, the occurrence of cardiovascular events in the SPKT group (*n* = 3, 7%) was significantly diminished after transplantation compared to that in the KTA recipients (*n* = 6, 28%; *p* = 0.02). The cardiovascular death rate was higher in KTA recipients (19%) than in SPK recipients with functioning grafts (3.3%) and comparable to that in patients with failed SPKT (16.7%) (*p* = 0.16).

In line with pretransplant values, SPKT recipients showed significant improvements in Hb1ac values (*p* = 0.001), blood pressure control (*p* =  < 0.005) and low-density lipoprotein/high-density lipoprotein (LDL/HDL) ratio (*p* =  < 0.005) 5 years after transplantation. With regard to echocardiographic assessment, SPKT recipients showed significant improvements in left ventricular systolic parameters during follow-up.

**Conclusions:**

Normoglycaemia and improvement of lipid metabolism and blood pressure control achieved by successful SPKT are associated with beneficial effects on survival, cardiovascular outcomes and systolic left ventricular cardiac function. Future studies with larger samples are needed to make predictions regarding cardiovascular events and graft survival.

**Supplementary Information:**

The online version contains supplementary material available at 10.1186/s12882-021-02522-8.

## Introduction

Diabetes mellitus (DM) remains the predominant risk factor for the development of chronic kidney disease (CKD), followed by end-stage renal disease (ESRD) [1]. The leading cause of death in patients with ESRD, both before and after transplantation, is cardiovascular disease (CVD) [2, 3].

CVD mortality in haemodialysis patients is tenfold higher than that in the general population [4]. The significantly increased risk is explainable through overrepresentation of arteriosclerosis risk factors. These risk factors include arterial hypertension caused by dysregulation of the renin–angiotensin–aldosterone system (RAAS) and electrolyte imbalance due to failing renal function. In diabetic ESRD, dyslipidaemia and a body mass index (BMI) > 25 kg/m^2^ are additional risk factors for developing CVD and subsequent increased morbidity and mortality. Researchers suggest that this increase is related to diffuse peripheral coronary arteriosclerosis and higher left ventricular mass in diabetic patients compared to controls [5, 6]; moreover, diabetes mellitus can cause functional and structural changes in the myocardium without coronary artery disease, a disorder known as diabetic cardiomyopathy (DCM) [7]. Furthermore, diastolic filling is frequently impaired, and hypertension is a major problem.

For patients with ESRD, kidney transplantation is, in general, the treatment of choice, offering improved survival and reduced morbidity and mortality compared with continuous dialysis [8]. However, in patients with both insulin-dependent type 1 diabetes and ESRD, simultaneous pancreas and kidney transplantation (SPKT) is the gold standard. The obvious benefits of functioning pancreas transplantation include the normalization of blood glucose without the use of insulin and the removal of uraemia, the hyperglycaemic state of diabetes, and the positive macro- and microvascular effects.

However, cardiovascular complications and CAD remain the predominant causes of death following successful kidney transplantation [2, 3]. The question of whether cardiovascular outcomes and cardiac function can be improved by SPKT or kidney transplantation alone (KTA) is important and is still debated. Most recent studies on cardiovascular outcome focused on patients with DM I showing significantly better results in SPKT compared to KTA (exclusively living kidney transplantation) [9, 10].

Due to the lack of organs, local center policies such as (inter-)national allocation guidelines there has been a tendency to select younger and healthier DM I patients with ERDS to SPKT, and older DM (I)/II recipients with more comorbidities to KTA. Thus, to overcome the resulting imbalance of a direct pre- and posttransplant patient comparison, we performed propensity score matching (PSM) to evaluate cardiovascular outcomes between SPKT and KTA- recipients.

The purpose of our retrospective study was to assess, with a PSM, the effect of long-term normoglycaemia—as achieved by successful SPKT—on long-term cardiovascular function and outcomes in IDMD patients compared to KTA. Furthermore, we wanted to use cardiac diagnostics and echocardiography to determine whether there is a relationship between systolic and diastolic dysfunction and metabolic control in diabetic patients.

## Patients and methods

### Study population and study design

The study protocol was approved by the local ethics committee of the University of Leipzig [AZ: Nr: 111–16-14032016]. From a prospectively collected electronic database, we retrospectively analysed medical data from all patients with IDDM undergoing SPKT and KTA at the University Hospital of Leipzig between 1998 and 2019 with special emphasis on treatment-related cardiovascular function and outcome. The exclusion criteria consisted of patients younger than 18 years, pancreas retransplantation, and those with missing data.

### Outcome analysis

In addition to patient and graft characteristics, a main focus was placed on metabolic outcomes, especially on cardiovascular and arteriosclerotic risk factors, cardiac function and cardiovascular outcome. Standard demographic and clinicopathological characteristics were collected and analysed before, at the time of and after transplantation (in the follow-up period) for each patient: pretransplant data including recipient and donor characteristics such as age, gender, body mass index (BMI) and additional data including duration of diabetes mellitus (years), smoking habits, time of the waiting list, duration of pretransplant dialysis, endocrine and lipid metabolism (HbA1c, C-peptide, LDL/HDL ratio). Cardiovascular disease and risk factors included information about the cardiovascular system, such as the presence of CVD (coronary artery bypass graft/stent), cerebrovascular accident (CVA), peripheral arterial occlusive disease (PAOD), blood pressure parameters and the number of antihypertensive agents.

Peri- and postoperative data included information on immunosuppressive therapy as well as patient and graft function.

### Evaluation and assessment of the cardiovascular system

All patients with IDDM who were potential transplant candidates underwent structured cardiovascular examinations with echocardiography and coronary angiography (and intervention when necessary) as a routine part of the cardiac work-up at our centre before inclusion on the waiting list. For the assessment of PAOD or carotid artery stenosis, a structured algorithm with ultrasound and/or computed (CT)-/magnetic resonance (MR)-angiography was performed.

Assessments of cardiovascular outcomes posttransplant were performed yearly in all patients by a detailed and structured cardiovascular work-up including physical examination, echocardiography, electrocardiogram, duplex sonography of the carotid arteries and leg arteries such as chest-x ray and blood pressure values.

Cardiovascular, cerebrovascular and peripheral vascular events were regularly recorded during follow-up. CVD was defined as coronary artery luminal diameter stenosis at or above 50% in at least one segment, using a 16-segment American Heart Association coronary artery classification [11], with or without revascularisation, including percutaneous coronary intervention and/or coronary artery bypass grafting, and/or having a myocardial infarction. Cerebrovascular events were defined as having symptoms and/or radiographic findings consistent with stroke. PAOD was defined as intermittent claudication and/or the need for surgical or interventional procedures in the lower extremities.

Furthermore, the systolic and diastolic cardiac function of our patients was evaluated by regularly undergoing echocardiography. In this context, we obtained Doppler echocardiographic images using digital high-end cardiac ultrasound scanners. Conventional parasternal and apical imaging views were used to obtain left ventricular (LV) ejection fraction (LVEF), LV fractional shorting (LVFS), systolic LV dimension (LVDs), diastolic LV dimension (LVDd) and LV posterior wall diastole (LVPWd), and interventricular septal thickness in diastole (IVSd). Left ventricular mass (LVM) was calculated according to the Devereux formula and indexed for body surface area giving LVM index (LVMI) [12]. Furthermore, we determined LV hypertrophy, hypokinesia and compliance. All echocardiogram reports were acquired, recorded and classified using an American Society of Echocardiography-recommended scanning protocol with standard techniques [13].

### Surgical technique/immunosuppression

The procurement and transplantation of pancreas and kidney allografts were performed according to international standards and guidelines, as previously described [14–17]. The standard immunosuppression protocol of our centre consisted of induction therapy followed by triple maintenance medical therapy, as described previously [18].

### Statistical analysis

Continuous variables are reported as the mean ± SD and absolute numbers. Categorical data are described using frequencies. Student’s t-test for independent samples or the Mann–Whitney U (Wilcoxon) test were used to compare continuous variables as appropriate. Categorical variables were compared with the use of the Pearson χ2 test, Fisher’s exact test (was applied if the number of observations per cell was fewer than 5) and McNemar’s test as appropriate.

Patients were stratified according to the type of operative procedure, and PSM analysis was performed to match patients who underwent SPKT with patients treated with KTA. This allowed for a significant reduction in differences in baseline characteristics between the two patient cohorts, thus minimizing the impact of treatment-related selection bias.

To compute propensity scores, a logistic regression model was performed including the following known cardiovascular matching parameters: patient age, sex, body mass index, type of diabetes, duration of diabetes mellitus, prevalence of pretransplant dialysis, presence of coronary heart disease, PAOD and smoking history.

Predicted probabilities of belonging to a group based on this model were used as propensity scores. Based on these scores, 2:1 propensity score matching on implant techniques (SPKT versus KTA) using the nearest neighbour method was performed.

The calliper was set to 0.1 to ensure that only patients with similar propensity scores were matched. After matching, differences within categorical variables were calculated by the McNemar test, and continuous variables were calculated by the Mann–Whitney U (Wilcoxon) test.

The primary end point of this study included cardiovascular outcomes/echocardiographic findings such as all cause mortality. The secondary end point included graft survival. Pancreatic graft failure was defined as insulin substitution, retransplantation, patient death or loss of follow-up return to transplant. Kidney graft failure was defined as the need for dialysis, retransplantation, patient death or loss of follow-up.

Survival differences were assessed for statistical significance by Kaplan–Meier analysis and log-rank test. Cox proportional hazard regression models were used to calculate hazard ratios (HRs) for patient death in relation to treatment modality.

The association between the type of treatment and mortality was then assessed after adjustment for known cardiovascular risk factors, including recipient age, sex, recipient BMI, duration of diabetes mellitus, smoking habits, duration of dialysis, aspirin and statin use and known cardiovascular comorbidity (heart failure or CAD, CVA and MI).

Statistical analyses were conducted using IBM SPSS Statistics for Windows, version 24 (IBM, Armonk, NY). For all analyses, a *p*-value < 0.05 was considered statistically significant.

## Results

### Baseline patient characteristics

Of 127 patients receiving SPKT (*n* = 101) and KTA (*n* = 26) at our centre, 62 patients (*n* = 48,8%) remained after PSM. Of these, in 42 patients (*n* = 67,7%), SPKT was performed, and 21 (*n* = 50%) underwent KTA. The donor, recipient and pretransplant baseline characteristics of the study population are shown in Table 1, and the mean follow-up time after transplantation was 7.7 ± 3.95 years.

**Table 1:** Patient’s characteristics

Variable	SPKT (*n* = 42)	KTA (*n* = 21)	p
**Median follow-up time *(years)***	6.74 ± *5.3*	6.67 ± *2.6*	0.954
**Recipient Characteristics**			
** Age at transplantation (years)**	49.1 ± *4.7*	51.3 ± *3.2*	0.789
** Male Gender**	28 *(66.7%)*	13 *(61.9%)*	0.709
** Duration of diabetes *(years)***	28.6 ± *7.3*	27.1 ± *6.1*	0.671
** Type of diabetes**			
* type 1*	29 *(69%)*	12 *(57%)*	**0.173**
* type 2*	13 *(21%)*	9 *(33%)*	
**Living kidney donation**		7 (33.3%)	-
**Dialysis pretransplant**	38 *(90%)*	19 *(90.5%)*	1.0
**Dialysis duration pretransplant *(months)***	34.74 ± *32.2*	54.8 ± *46.3*	0.128
**Waiting time pretransplant (*months)***	11.2 ± *2.3*	15.4 ± *5.3*	0.378
**Coronary heart disease**	26 *(62%)*	16 *(76%)*	0.257
one-vessel coronary artery disease	13 *(31%)*	7 *(33%)*	0.848
two-vessel coronary artery disease	6 *(14.3%)*	3 *(14.3%)*	0.775
three-vessel coronary artery disease	6 *(14.3%)*	6 *(28.6%)*	0.173
coronary stents	12 *(29%)*	9 *(42%)*	0.257
* Peripheral arterial occlusive disease*	13 *(31%)*	8 *(38%)*	0.571
**Pre-transplant amputations**	7 (17%)	4 (19%)	0.814
**Arterial hypertension history *(years)***	11,4 ± *7.1*	16.8 ± *11.1*	0.208
**Nicotine abuse**	13 *(31%)*	7 *(35%)*	0.845
**Aspirin pretransplant**	17 *(42%)*	16 *(76%)*	**0.01**
**Statin pretransplant**	28 *(68%)*	16 *(76%)*	0.338
**Donor Characteristics**			
** Age (years)**	28.2 ± *11.5*	50.8 ± *13.5*	**˂0.01**
** Male donor**	26 (62%)	9 (43%)	0.151
** Donor BMI**	22.8 ± *2.7*	24.6 ± *2.92*	0.09
** Donor cardiovascular cause of death**	25 (59%)	9 *(43%)*	0.211
** Cardiopulmonary resuscitation**	2 (4.8%)	1 (4.7%)	1.0
** Diabetes mellitus**	0 (0%)	1 (4.7%)	0.139
** Arterial hypertension**	4 (9.5%)	6 (29%)	0.06
** Sodium (mEq/L)**	145.5 ± 1.3	147 ± 2.1	0.345
*** Creatinine (ummol/L)***	77 ± 8.2	79.2 ± 6.2	0.755
*** Urea (mmmol/L)***	6.3 ± 1.5	7.1 ± 2.1	0.507
** Intensive care unit stay (days)**	3.9 ± 0.9	4.6 ± 1.2	0.581
** Cardiovascular events pretransplant**	10 *(23.8%)*	10 *(47.6%)*	0.06
myocardial infarction	3 *(7.1%)*	5 *(23.8%)*	0.06
* percutaneous coronary intervention*	4 *(9.5%)*	3 *(14.2%)*	0.571
* coronary artery bypass graft*	3 *(7.1%)*	2 *(9.5%)*	0.741
** Cardiovascular events posttransplant**	3 *(7%)*	5 *(23%)*	**0.05**
* myocardial infarction*	2 *(4.7%)*	2 *(10%)*	0.464
* percutaneous coronary intervention*	1 *(2.3%)*	3 *(13%)*	0.067
* coronary artery bypass graft*	0	0	1.0
** Cerebrovascular accident pretransplant**	2 *(4.8%)*	2 *(9.5%)*	0.465
** Cerebrovascular accident posttransplant**	1 (2.4%)	3 *(14.3%)*	0.06
** Death**	9 *(21%)*	9 *(42.8%)*	0.08
** Cardiovascular cause of death**	3 *(7.1%)*	3 *(14.2%)*	0.363

Data are presented as the mean ± standard deviation and percentages of the total (%)

In the SPKT group, the donor age (*p* < 0.05) and the pretransplant use of aspirin (*p* = 0.01) were significantly lower. Furthermore, patients in the KTA group tended to have more pretransplant CVE (*p* = 0.06), as well as the donor BMI (*p* = 0.09) and the rate of donor hypertension (*p* = 0.06) were slightly higher. Otherwise, there were no significant pretransplant differences between the groups.

### Postoperative outcome, graft function and metabolic outcome

Regarding postoperative long-term outcome parameters slightly increased rates of delayed graft function (DGF) (KTA: 6 (29%) versus SPKT: 5 (12%); *p* = 0.09) and proteinuria (KTA: 8 (38%) versus SPKT: 7 (16%); *p* = 0.06) could be observed in the KTA group compared to SPKT recipients. (Supplementary file 1).

With regard to HbA1c levels, significant differences were observed preoperatively between the two groups (*p* < 0.01) (Table 2). As expected, the values decreased significantly posttransplant in SPKT recipients, whereas in the KTA- group, the pretransplant HbA1c value was lower but deteriorated markedly during the follow-up course compared to pretransplant values. Significant differences between both groups were observed at 3 and 5 years after transplantation (at 3 years: SPKT 5.6% versus KTA 6.9%; *p* = 0.001; at 5 years: SPKT 5.9% versus KTA 7.3%; *p* = 0.001).

**Table 2:** Metabolic outcome

**Variable**	**SKPT**	**P**^**1**^	**KTA**	**P**^**1**^	**P**^**2**^
**HbA1c in %**					
* pretransplant*	7.4 ± 1.5		6.2 ± 0.9		**0.001**
* follow-up 1 year*	5.8 ± 1.2	**˂0.05**	6.6 ± 1.3	0.01	0.062
* follow-up 3 years*	5.6 ± 1.1	**˂0.05**	6.9 ± 1.8	0.01	**0.001**
* follow-up 5 years*	6.7 ± 1.4	**˂0.05**	7.3 ± 1.9	< 0.005	**0.001**
**GFR (ml/min)**					
* pretransplant*	13.61 ± *11.06*		12.95 ± *6.54*	**0.01**	0.596
* follow-up 1 year*	51.33 ± *14.4*	** < 0.005**	33.7 ± *14.81*	**0.04**	**0.001**
* follow-up 3 years*	57.5 ± *15.3*	** < 0.005**	30.2 ± *14.8*	**0.001**	**0.008**
* follow-up 5 years*	52.3 ± *12.9*	** < 0.005**	35.2 ± *11.2*	0.1	**0.029**
**BMI in kg/m2**					
* pretransplant*	27.8 ± 4.3		27.8 ± 3.3		0.692
* follow-up 1 year*	26.5 ± 4.7	0.135	29.4 ± 3.6	0.143	**0.025**
* follow-up 3 years*	26.6 ± 3.8	0.196	29.3 ± 3.6	0.181	0.307
* follow-up 5 years*	27.1 ± 4.7	0.678	28.9 ± 2.6	0.190	0.471
**LDL/HDL-ratio**					
* pretransplant*	2.3 ± 1.2		2.2 ± 0.9		0.96
* follow-up 1 year*	2.0 ± 1.3	0.09	2.6 ± 0.9	< 0.005	**0.042**
* follow-up 3 years*	1.8 ± 1.1	** < 0.005**	2.7 ± 0.9	< 0.005	**0.02**
* follow-up 5 years*	1.9 ± 0.9	** < 0.005**	2.8 ± 0.9	< 0.005	**0.04**

data are presented as the mean ± standard deviation

P^1^ = intra-group *p*-values; P^2^ = inter-group *p*-values

In the stable phase, 1, 3 and 5 years after transplantation, significant differences in the estimated glomerular infiltration rate (GFR) were observed between the transplant groups.

With regard to BMI, there were no significant differences between the two groups in the pretransplant values and at 3 and 5 years after transplantation; there was only a slight improvement in the SPKT group compared to a moderate deterioration for the KTA patients.

More evident is the improvement of LDL/HDL-ratio after SPKT transplantation in contrast to the KTA patients where a considerable worsening takes place. Even though the pretransplant values were not significantly different (*p* = 0.96), they showed a significant shift during the posttransplant course at 1, 3 and 5 years in opposite directions (Table 2).

### Survival

**Fig. 1:**
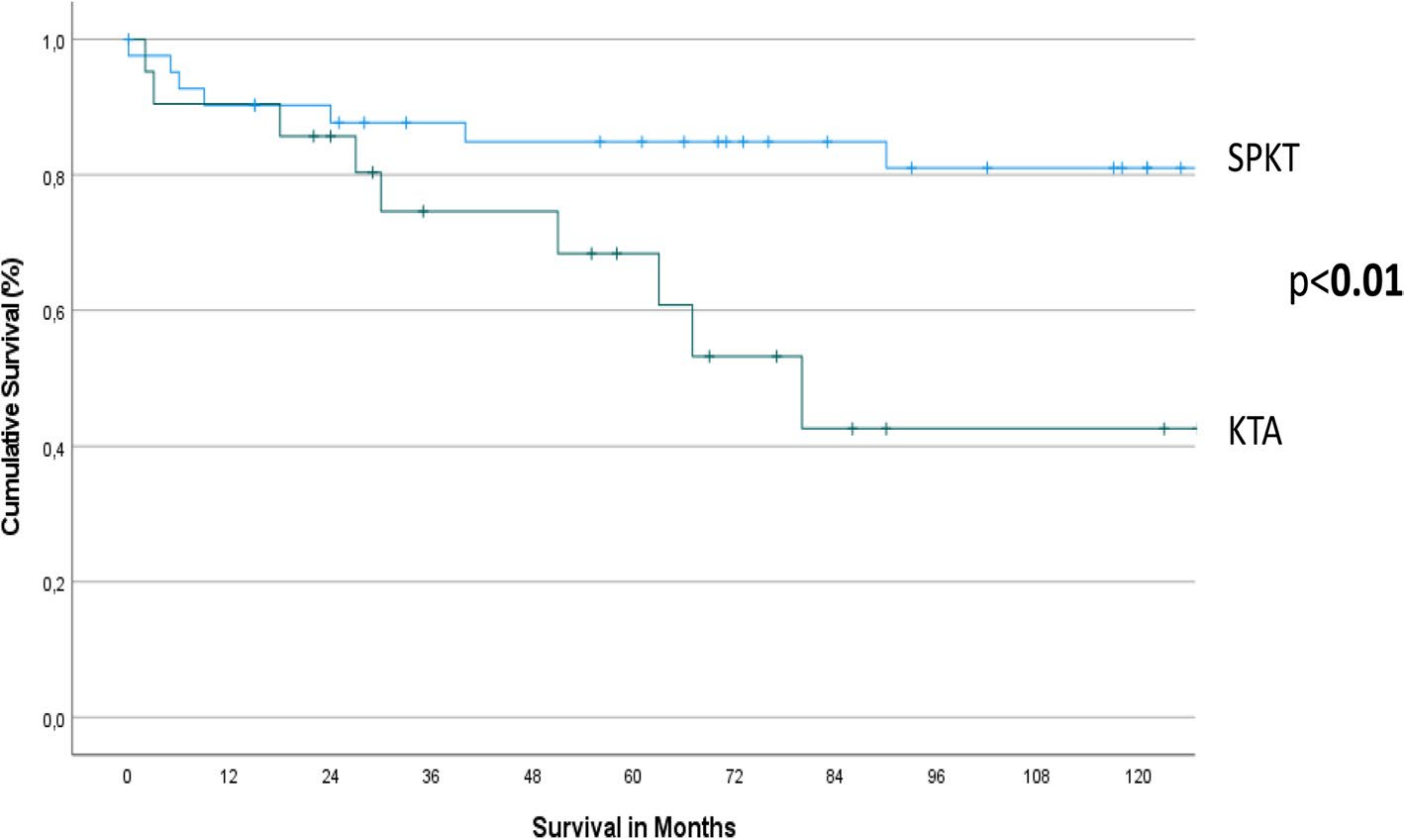
Patient Survival after simultaneous pancreas kidney transplantation (SPKT) and kidney transplantation alone (KTA)

The 1-, 3-, 5- and 10-year overall survival rates in patients after SPKT were 97.5%, 93%, 88% and 76%, respectively, and 95%, 85.5%, 76% and 52% after KTA (*p* < 0.01) (Fig. 1).

The 1-, 3-, 5- and 10-year pancreas graft survival rates were 83.1%, 77.3%, 74.1% and 70.2%, respectively.

Kidney graft survival rates were 85.1%, 80%, 73.4% and 68.5% for the SPKT group and 81%, 67.2%, 51% and 27% for the KTA group, respectively (*p* = 0.03). Cardiovascular death rates were more evident in the KTA group (*n* = 3; 14.1%) than in the SPKT group (*n* = 3; 7.1%; *p* = 0.363).

In multivariate Cox regression analysis, after adjustment for patient age, sex, type of diabetes, prevalence of pretransplant dialysis, presence of coronary heart disease, PAOD and smoking history, the HR for mortality in SPKT recipients was 0.63 (95%CI: 0.49–0.82; *p* < 0.05), using KTA recipients as a reference. Additionally, cardiovascular comorbidity could be identified as a significant independent predictor for mortality (HR 1.6, 95%CI: 1.21–2.1; *p* < 0.05). (Table 3).

**Table 3:** Cox regression analysis for death in SPKT recipients

Variable	HR	95%CI	*p*-value
Transplant Type		0.49–0.82	** < 0.05**
KTA	Reference		
SPKT	0.63		
Recipient age (*years)*	0.97	0.93–1.01	0.215
Gender	1.13	0.31–4.1	0.851
Diabetes duration (*years)*	1.03	0.92–1.08	0.932
Number of antihypertensive drugs	0.97	0.82–1.12	0.564
Cardiovascular comorbidity	1.6	1.21–2.1	** < 0.05**
Smoking history	1.28	0.95–1.7	0.1
Dialysis duration (*months)*	1.08	0.99–1.01	0.165

Data are presented as absolute numbers

### Cardiovascular outcomes

The data obtained at baseline and follow-up are presented in Tables 1 and 4.

**Table 4:** Cardiovascular outcome

**Variable**	**SKPT**	**P**^**1**^	**KTA**	**P**^**1**^	**P**^**2**^
**arterial hypertension posttransplant, n**	39 *(93%)*		20 (95%)		0.715
**MAD in mmHG**					
* pretransplant*	101.2 ± *10.9*		96.3 ± *11.1*		0.09
* follow-up 1 year*	91.6 ± *5.4*	**˂0.005**	98.7 ± *9.1*	0.161	**0.001**
* follow-up 3 years*	90.8 ± *10.7*	** < 0.005**	100.1 ± *5.2*	**0.06**	**0.001**
* follow-up 5 years*	88.9 ± *11.8*	** < 0.005**	101.4 ± *3.2*	**0.01**	**0.001**
**Number of antihypertensive medications, n**	2.67 ± 1.7		2,29 ± 1.8		
** > 3 antihypertensive medications, n**	12 *(28%)*		5 *(23%)*		0.788
**Cardiovascular events post-transplant**	3 *(7%)*		5 *(23%)*		**0.05**
* myocardial infarction*	2 *(4.7%)*		2 *(19%)*		0.464
* Percutaneous coronary*	1 *(2.3%)*		3 *(13%)*		0.067
* intervention*					
* Coronary artery bypass*	0		0		1.0
***Cerebrovascular accident posttransplant***	1 *(2.4%)*		3 *(14.3%)*		0.06
***Statin at follow-up***	17 (42%)		13 (61%)		**0.048**
***Aspirin at follow-up***	26 (62%)		15 (71%)		**0.045**

data are present as the mean ± standard deviation or as absolute numbers (n) and percentages of the total (%)

P^1^ = intra-group *p*-values; P^2^ = inter-group *p*-values

Mean MAP recordings between SPKT recipients and KTA patients were comparable before transplantation (SPKT: 101.2 ± *10.9* mmHg versus KTA: 96.3 ± *11.1 mmHg*; *p* = 0.09). During follow-up, significant differences were seen at 1 year (SPKT: 91.6 ± 5.4 mmHg versus KTA: 98.7 ± 9.1 mmHg; *p* = 0.001), 3 years (SPKT: 90.8 ± 10.7 mmHg versus KTA: 100.1 ± 5.2 mmHg; *p* = 0.001) and 5 years (SPKT: 88.9 ± 11.8 mmHg versus KTA: 101.4 ± 3.2; *p* = 0.001) after transplantation. This means significant improvements in MAP values in the SPKT recipients (preoperative versus 5 years: *p* < 0.01) compared to significant deterioration in the KTA group (preoperative versus 5 years: *p* = 0.01).

A significantly lower rate of CVE (SPKT: *n* = 3 (7.1%) versus KTA: *n* = 6 (28%); *p* = 0.02) was observed in the posttransplant follow-up in SPKT patients than in KTA patients (Table 4). Furthermore, in the KTA group, a trend towards higher rates of CVA was seen after transplantation compared to SPKT patients (*p* = 0.06).

Additionally, the posttransplant use of aspirin (*p* = 0.048) and statin medications (*p* = 0.042) was significantly lower in the SPKT group than in the KTA group.

No differences were shown for the rate of hypertensive patients or the number of antihypertensive medications between SPKT and KTA recipients. With regard to survival, cardiovascular long-term outcome and events similar results could be observed in sub-group analysis between KTA- and SPKT-failed recipients (Supplementary file 2).

### Echocardiographic findings

Echocardiographic data and findings pretransplant and in the follow-up of both study groups are presented in Table 5. Significant improvements concerning SPKT patients from pre- to posttransplant values could be observed with regard to most of the left ventricular structure values and systolic/diastolic cardiac function parameters. Specifically, we observed significant posttransplant differences in LVDs (*p* = 0.04), LVDd (*p* = 0.011), LVMI (*p* = 0.04) and LVFS (*p* = 0.021) between SPKT and KTA recipients.

**Table 5:** Echocardiographic characteristics of the study patients

Variable	SPKT	P^1^	KTA	P^1^	P^2^
**LV structure**					
** LVPWd in mm**					
* pretransplant*	12.4 ± *2.9*		13.8 ± *2.6*		0.06
* follow-up*	13.8 ± *3.1*	0.176	14.7 ± *2.6*	0.234	0.261
** IVSd in mm**					
* pretransplant*	13.8 ± *2.8*		13.1 ± *3.3*		0.145
* follow-up*	12.4 ± *2.9*	0.07	13.3 ± *2.5*	0.789	0.765
** LVDd in mm**					
pretransplant	48.7 ± *4.2*		49.8 ± *6.8*		0.754
follow-up	45.3 ± *5.2*	**0.016**	51.8 ± *9.6*	0.43	**0.011**
** LVDs in mm**					
pretransplant	32.5 ± *6.2*		30.9 ± *4.2*		0.823
* follow-up*	28.1 ± *5.1*	**0.05**	32.1 ± *5.2*	0.67	**0.04**
** LVMI in mm**					
pretransplant	92.1 ± *16.9*		98.6 ± *15.4*		0.21
follow-up	82.3 ± *23.4*	**0.01**	89.4 ± *10.2*	0.1	**0.04**
**LV systolic function**					
** LVEF in %**					
* pretransplant*	59.8 ± *9.1*		53.1 ± *7.9*		**0.017**
* follow-up*	64.5 ± *8.5*	**0.035**	57.8 ± *8.3*	0.159	**0.013**
** LVFS in %**					
pretransplant	35.1 ± *7.6*		31.9 ± *6.8*		0.369
* follow-up*	41.2 ± *8.8*	**0.02**	35.1 ± *7.9*	0.424	**0.021**

data are present as the mean ± standard deviation; P^1^ = intra-group *p*-values; P^2^ = inter-group *p*-values

*LV* left ventricular, *LVPWd* LV posterior wall diastole, *IVSd* interventricular septum diastole, *LVDd* diastolic LV dimension, *LVDs* systolic LV dimension, *LVMI* LV mass index, *LVEF* LV ejection fraction, *LVFS* LV fractional shorting

Furthermore, in Table 6, we analysed the changes in echocardiographic function, structure and morphology during the study period between SPKT- and KTA recipients. There was an amelioration in LV function and significant LV hypertrophy in the SPK group and a deterioration of normal status to high-grade hypertrophy in the KT group. In addition, there was a significant improvement in hypokinesia in the SPKT group, whereas hypokinesia posttransplant occurred more often in the KT group.

**Table 6:** Changes in echocardiographic function, structure and morphology during the study period

**Variable**	**SPKT**			**KTA**		
	**pre**	**post**	***P*-value**	**pre**	**post**	***P*-value**
**LV ejection fraction (%)**						
normal (< 50%)	33 *(79%)*	37 *(88%)*	1.00	15 *(71%)*	13 *(63%)*	0.90
slightly reduced (< 40–50%)	7 *(17%)*	4 *(9.3%)*	1.00	4 *(19%)*	5 *(24%)*	1.00
moderately reduced (> 30–40%)	2 *(5%)*	1 *(2.7%)*	0.39	2 *(10%)*	2 *(9%)*	0.62
reduced (< 30%)	0	0	1.00	0	1 *(5%)*	0.89
**LV hypertrophy (%)**						
normal (♀49–115 g/m2♂43–95 g/m2)	12 (*28.6*%)	18 (*42.9*%)	0.40	8 *(40%)*	3 *(15.8%)*	0.06
slightly (♀116–131 g/m2♂96–108 g/m2)	18 (*42.9*%)	17 (*40.5*%)	0.26	8 *(40%)*	9 *(42.1%)*	0.39
moderately (♀132–148 g/m2♂109–121 g/m2)	9 (*21.4*%)	5 *(11.9%)*	**0.05**	3 *(15%)*	4 *(21.1%)*	0.40
high-grade (♀ > 149 g/m2♂ < 122 g/m2)	3 *(7.1%)*	2 (*4.8*%)	0.06	1 *(5%)*	4 *(21.1%)*	**0.05**
**Hypokinesia (%)**	15 (*38*%)	7 (*20*%)	**0.005**	5 *(23.8%)*	9 *(53%)*	0.1
**Compliance (%)**	23 *(58.5%)*	15 *(39%)*	**0.09**	8 *(44%)*	10 *(56%)*	0.328

Data are presented as absolute numbers (n) and cumulative percentages (%)

*pre* pretransplant, *post* posttransplant, *LV* left ventricular

## Discussion

The severe impact of diabetes on cardiovascular morbidity and mortality though micro- and macrovascular alterations is well known [19–21]. The study of *Gowdak *et al*.* concludes that diabetes confers a cardiovascular risk comparable to that of coronary artery disease (CAD) in transplanted patients without diabetes [22]. Patients on the waiting list suffer from stroke, coronary artery disease, congestive heart failure and arrythmia, but transplanted patients are diagnosed more often with cardiovascular disease than the general population [23, 24].

The present study examined the cardiovascular outcomes and function of a large cohort of patients who underwent SPKT or KTA for IDDM in a propensity score matching analysis manner. Our results demonstrate that normoglycaemia, achieved by successful pancreas transplantation, slows the progression of some of the features of macrovascular disease in IDDM patients, mainly blood pressure controls, lipid metabolism, the presence of cardio- and cerebrovascular events and cardiac function and performance, and indicates significant improvements in these parameters.

In accordance with currently available literature, we could observe that normoglycemia achieved by long-term successful SPKT in type 1 IDDM patients was associated with reduced all-cause mortality/ increased long-term patient survival compared to KTA [10, 25–32].

As shown by previous studies success of SPKT depends on long-term functioning pancreas grafts and pancreas graft loss is inversely associated with long-term survival [26, 33–35]. This is consistent with our findings of lower mortality in SPKT recipients with functioning pancreas grafts post-transplant compared with those of failing pancreas grafts.

Our initial cohort of 127 patients (100 SPKT/27 KTA) was heterogeneous in favour of younger, cardiovascularly healthier SPKT recipients as opposed to older, cardiovascularly multimorbid KTA patients who were more likely to have DM II.

However, by using propensity score matching on patient characteristics, including recipient age at transplantation, sex, type of diabetes mellitus, and prevalence of pretransplant dialysis, such as cardiovascular history, we were able to create a homogenous and similar study population upon entrance into the study. Therefore, it can be concluded that the different outcomes observed are consequences of the transplant. This conclusion could be confirmed by the observed results of our analyses in the subgroup of pancreas-kidney failure patients. This subgroup was closer to the KTA group with regard to survival, cardiovascular and metabolic outcomes.

There are actually only a few studies assessing cardiovascular outcomes and cardiac performance/function with regard to long-term follow-up in SPKT recipients compared to KTA recipients. *Biesenbach *et al*.* showed that the progression of macrovascular diseases was significantly lower in recipients with a functioning SPK graft than in KTA recipients [36]. *La Rocca *et al*.* detected fewer cardiovascular events and better survival after SPKT than after KTA [37].

Our results are also supported by the study of *Jukema *et al*. *[38], who compared the progression of atherosclerosis (measured by mean-segment diameter loss on coronary angiography) in 26 patients with and 6 patients without a functioning pancreas after SPKT transplantation after a mean follow-up time of 3.9 years. They observed a reduction in arteriosclerotic progression in patients with successful SKPT and achieved normoglycaemia compared to recipients with pancreatic graft failure. Indeed, the analysed use/frequency of antihypertensive/statin medications in our follow-up 5 years after transplantation with regard to our study groups further highlights the beneficial effect of SPKT on the progression of coronary arteriosclerosis.

On the other hand, our findings on better cardiovascular outcomes after SPKT compared with KTA are highlighted by the study of *Lindahl *et al. [39] which showed that in patients with DM I and ESRD, SPKT was associated with reduced long-term cardiovascular mortality compared with living donor kidney transplantation. The same study group examined later in a smaller collective if normoglycaemia achieved by successful SPKT could slow the long-term progression of CVD/reduce CVEs based on 10-year follow-up values when compared to living kidney transplantation. However, earlier results could not be confirmed in long-term follow-up because a detailed breakdown of values in short- and midterm follow-up was not performed, and as the group correctly noted, the selection of survivors resulted in a clear selection bias.

However, because of performing PSM and subgroup analysis of patients with failed SPKT, we demonstrated that observed significant improvements regarding both metabolic/cardiovascular outcome, cardiac function and survival were consequences of the different transplant types and better glucometabolic effects in the SPKT recipients.

CVD is the most common and the most common cause of death among diabetic recipients of KTA. Modern immunosuppressive medications, specifically CNIs and steroids, are known contributors to arteriosclerosis and represent CV risk factors due to associated hypertension, hyperlipidaemia and glucose tolerance. In this context, adequate steroid withdrawal or avoidance could reduce this risk. In the present analysis, we showed that 67% of patients in the SPKT group versus 55% of patients (*p* = 0.2) in the KTA group were steroid-free after 1 year.

Nevertheless, at our centre, target levels for calcineurin inhibitors (CNIs) and doses of steroids were historically higher in SPKT recipients than in KTA recipients. However, the hypothesis that different levels and immunosuppressive regimens between SPKT and KTA patients could be responsible for detrimental effects on CVD risk after transplantation could not be statistically supported by our data. In the past, it was already reported that successful SPKT with subsequent glucometabolic control has an impact on posttransplant systolic and diastolic cardiac function [29, 40].

Our echocardiographic analyses evaluated the development of different echocardiographic parameters with a special focus on left ventricular structural and functional (systolic and diastolic) dimensions before and after SPKT and KTA.

Based on our echocardiographic findings, we found significant improvements in most of the structural LV hypertrophy parameters in SPKT recipients and a deterioration of normal status to high-grade hypertrophy in the KTA patients. With regard to systolic function observed as EF and LVFS changes, significant improvements in SPKT patients versus stabilization/small ameliorations in KTA recipients were observed during follow-up. In addition, there was a significant improvement in hypokinesia in the SPKT group, whereas hypokinesia posttransplant occurred more often in the KT group.

In addition, small significant improvements in diastolic cardiac function could be confirmed in our findings by reduction of LVMI and compliance after transplantation for the SPKT group compared to KTA patients. Nevertheless, it is well known that metabolic alterations lead first to diastolic dysfunction rather than systolic alterations, and more than 30% of diabetic patients with diastolic problems have normal systolic function [41].

To evaluate diastolic dysfunction in our study, the analysis of more diastolic parameters, such as peak filling rate, time-to-peak filling rate, peak filling rate/peak ejection rate ratio, diastolic flow velocity, ratio of rapid filling and atrial filling velocity (E/A ratio), such as early diastolic mitral annulus velocity (E/e' ratio), is required. However, a summary of our findings, such as amelioration of glucometabolic and lipid metabolism as well as improvement of left ventricular systolic structure and function, supports our thesis that normoglycaemia obtained by successful SPKT has a protective influence on survival and cardiovascular outcomes in IDDM patients compared to KTA.

This is in accordance with the results of *La Rocca *et al*.* and *Fiorina *et al*.,* who found that SPKT in DM I patients results in a better glucometabolic pattern and blood pressure control and an improvement in left ventricular function and structure as well as a reversal of diastolic dysfunction compared to KTA [29, 31].

Finally, more (non)-invasive risk stratification systems, such as the coronary artery calcium score [42] (CAS) and further coronary angiographic findings, could be helpful tools in pretransplant screening for high-risk individuals and for prediction of outcomes following SPKT and/or KTA.

The limitations of our study are the small number of participants and the retrospective design of the study. The period of time regarding performed transplantation was over 20 years long (1998–2016), which means that the diabetic treatment of some of the patients took already place in the 1970s and 1980s and is therefore not comparable to our present standards and limits the transferability of the results.

However, despite of accurate PSM analysis for homogenous patient collectives, comparison between SPKT and KTA transplants are complicated by differences in recipients, donors such as peri-/post-transplant related characteristics. SPKT recipients are often younger, have fewer comorbidities and pre-transplant dialysis times and received younger deceased donor organs with better quality. However, it should not be forgotten that SPKT is associated with an increased risk of early perioperative surgical complications and consequently considerable rate of pancreas graft loss during the first weeks. These may be inversely associated with short-term patient survival. As could be confirmed by our results, however, in KTA, higher recipient ages and donors with worse quality/ characteristics (e.g. increased ages and creatinine values…) consecutively resulting in worse post-transplant events (increased DGFs, proteinuria…) – have been accepted in recent years and might have lead to negative effects on survival and long-term outcome. However, it is difficult and in ways not amenable to adjust for all these inter-group differences and changes over time by analysing smaller sub-cohorts due to loss of statistical power. Despite of PSM, these alterations may introduce a bias with regard to better outcomes in favour of SPKT recipients.

On the other hand, due to general (inter-)national allocation criterias and guidelines as well as local center policy/protocols an age bias that younger deceased donors were used for SPKT grafts or recipients selected for SPKT must be less than 55 years of age (no formal upper age limit for KTA) is unavoidable. Unfortunately, subgroup analysis for e.g. donor age or donor comorbidities is due to low statistical power in our case not possible. In summary, there is a limited power of the study, with an increased risk of not disclosing a true treatment effect.

However, the strengths of this study include the long follow-up period and the largely homogenous study collective as a consequence of PSM. Our pretransplant cardiac evaluation was completed, and the postoperative follow-up by clinical examination and objectifiable values (e.g., laboratory results, echocardiography) were detailed and comprehensible.

## Conclusions

Our study shows in a PSM analysis manner that patients who achieved normoglycaemia by successful SPKT have increased patient survival compared to patients who underwent KTA. This difference was explained by the low prevalence of cardiovascular and cerebrovascular events in the SPKT group as a result of increased posttransplant cardiovascular function/performance with enhanced ejection fraction, improvement of left ventricular function and reversal of diastolic dysfunction, such as better blood pressure control and lipid metabolism.

However, further studies with larger patient numbers are necessary to evaluate the effects of preoperative identification of individuals at high coronary risk on long-term post-transplant function, cardiovascular outcomes and survival rates.

## Supplementary Information

**Additional file 1.**

**Additional file 2.**

## Data Availability

Our database contains highly sensitive data that may reveal clinical and personnel information about our patients and lead to their identification. Therefore, according to organizational restrictions and regulations, these data cannot be made publicly available. Nevertheless, the datasets used and/or analyzed in the current study are available from the corresponding author upon reasonable request.

## Abbreviations

ARF: Arteriosclerotic risk profile
BMI: Body mass index
CAD: Coronary artery disease
CKD: Chronic kidney disease
CNI: Calcineurin inhibitors
CVA: Cerebrovascular accident
CVD: Cardiovascular disease
CVE: Cardiovascular events
DM I: Diabetes mellitus type 1
DM II: Diabetes mellitus type II
DCM: Diabetic cardiomyopathy
ESRD: End-stage renal disease
HR: Hazard ratio
IDDM: Insulin dependent diabetes mellitus
IVSd: Interventricular septum diastole
KTA: Kidney transplant alone
LDL/HDL: Low-density lipoprotein/high-density lipoprotein
LV: Left ventricular
LVDd: Diastolic LV dimension
LVDs: Systolic LV dimension
LVEF: LV ejection fraction
LVFS: LC fractional shorting
LVPWd: LV posterior wall diastole
LVM: Left ventricular mass
LVMI: Left ventricular mass index
MAP: Mean arterial pressure
POST: Posttransplant
PRE: Pretransplant
PAOD: *peripheral arterial occlusive disease*
RAAS: Renin–angiotensin–aldosterone system
SPKT: Simultaneous pancreas kidney transplantation
PSM: Propensity scoring matching
CMV: Cytomegalovirus
