# Supplementary material for: Cardiovascular outcomes after simultaneous pancreas kidney transplantation compared to kidney transplantation alone: a propensity score matching analysis
Source: BMC Nephrol. 2021 Oct 21;22:347. doi: 10.1186/s12882-021-02522-8 (PMC8529792; doi:10.1186/s12882-021-02522-8)
Supplement: Supplementary file 1 — Additional file 1. [file 12882_2021_2522_MOESM1_ESM.docx]

**Supplementary File 1:** Postoperative outcome between SPKT- and KTA recipients

| **Variables** | **SPKT (n = 42 patients)** | **KTA (n = 21 patients)** | **p-value** |
| --- | --- | --- | --- |
| Cumulative 1-year rejection episode | 7 (19%) | 7 (33%) | 0.204 |
| CMV- Infection | 16 (38%) | 6 (29%) | 0.454 |
| Proteinuria/ Albuminuria | 7 (16%) | 8 (38%) | 0.060 |
| Delayed graft function renal | 5 (12%) | 6 (29%) | 0.090 |
