# Supplementary material for: Cardiovascular outcomes after simultaneous pancreas kidney transplantation compared to kidney transplantation alone: a propensity score matching analysis
Source: BMC Nephrol. 2021 Oct 21;22:347. doi: 10.1186/s12882-021-02522-8 (PMC8529792; doi:10.1186/s12882-021-02522-8)
Supplement: Supplementary file 2 — Additional file 2. [file 12882_2021_2522_MOESM2_ESM.docx]

**Supplementary File 2:** Cardiovascular outcomes and survival between the three patient groups

|  | **Patients** | **Survival/ All cause mortality** | **Cardiovascular Death Rate** | **Cardiovascular Events** |
| --- | --- | --- | --- | --- |
| SPKT | 30 | 4 /30 (13.3%) | 2/30 (6.5%) | 1/30 (5.5%) |
| KTA | 21 | 9 /21 (42%) | 3/21 (14.2%) | 5/21 (23.8%) |
| SPKT failed | 12 | 5/12 (42%) | 1/12 (8.3%) | 2/12 (16.7%) |
| p -value |  | 0.038 | 0.651 | 0.087 |
